# Successful Treatment of Large Left Ventricular Thrombosis During Extracorporeal Membrane Oxygenation (ECMO): A Case Report and Review of the Literature

**DOI:** 10.1002/ccr3.70123

**Published:** 2025-01-21

**Authors:** Xue‐ting Yang, Yi‐jin Chen, Han Zeng, Lei Deng, Li Chang, Yi Li, Meng‐long Song, Yang Zhang, Wei Peng, Dong Wang, Hua Jiang, Hong‐qiong Peng

**Affiliations:** ^1^ Emergency Intensive Care Unit Sichuan Provincial People's Hospital, University of Electronic Science and Technology of China Chengdu Sichuan China; ^2^ Institute for Emergency and Disaster Medicine, Sichuan Academy of Medical Science Sichuan Provincial People's Hospital, School of Medicine, University of Electronic Science and Technology of China Chengdu Sichuan China; ^3^ Emergency General Hospital Chaoyang District Beijing China; ^4^ Department of Emergency The First People's Hospital of Longquanyi District Chengdu Chengdu Sichuan China

**Keywords:** anticoagulant, circulatory failure, extracorporeal membrane oxygenation (ECMO), fulminant myocarditis, hemorrhagic events, left ventricular (LV) thrombus

## Abstract

We report a rare complication of left ventricular giant thrombus in a patient with fulminant myocarditis after venoarterial extracorporeal membrane oxygenation therapy. This case report offers simple anticoagulant treatment experiences to eliminate significant LV thrombosis in patient undergoing extracorporeal membrane oxygenation, so that patients do not need surgery.

## 1 Introduction

Acute fulminant myocarditis is a serious condition with rapid progression and with high fatality rate [1, 2]. It can lead to cardiac decompensation, circulatory failure, and respiratory failure in a short period of time. If not treated in time, patients may die from arrhythmias and sudden cardiac death within hours to days [3]. Extracorporeal membrane oxygenation (ECMO) is the first choice for the treatment of patients who suffer from acute cardiac failure with or without respiratory failure, especially for the rescue therapy of cardiac arrest patients [4]. As early as 50 years ago [5], it was the first successful attempt to apply ECMO technology to provide basic life support for a severe shock patient. After continuous improvement, it become a portable extracorporeal mechanical auxiliary device that is easy to operate and can provide longer life support. Subsequently, ECMO has been increasingly used in the rescue of critical diseases as a mechanical circulatory respiratory support device. According to different paths of blood transfusion, ECMO technology has two forms: veno‐arterial extracorporeal membrane oxygenation (VA‐ECMO) and veno‐venous extracorporeal membrane oxygenation (VV‐ECMO) [6]. The former can provide both circulatory and respiratory assistance while the latter only provides respiratory assistance. There is a widespread consensus that ECMO treatment should be more aggressively initiated in patients with both hemodynamic instability and fulminant myocarditis, as early as possible.

Thrombosis and hemorrhagic events represent the most prevalent complications associated with ECMO, and the incidence of thrombosis is as high as 52% [7]. Furthermore, the thrombus within an external conduit can potentially be pumped into the systemic circulation, leading to a variety of embolic complications [8, 9], including myocardial infarction, cerebral infarction, and pulmonary embolism. It is also the primary reason for ECMO treatment failure; once detected, thrombectomy should be intervened immediately. However, patients with fulminant myocarditis hardly tolerate the surgical intervention, and frequently face an extremely high risk of death. In order to offer valuable insights into clinical ECMO treatment, particularly regarding anticoagulation management and rescue treatment after thrombosis. This article reports a case of left ventricular (LV) thrombosis successfully treated with simple anticoagulant strategy during ECMO treatment.

## 2 Case Presentation

**FIGURE 1:**
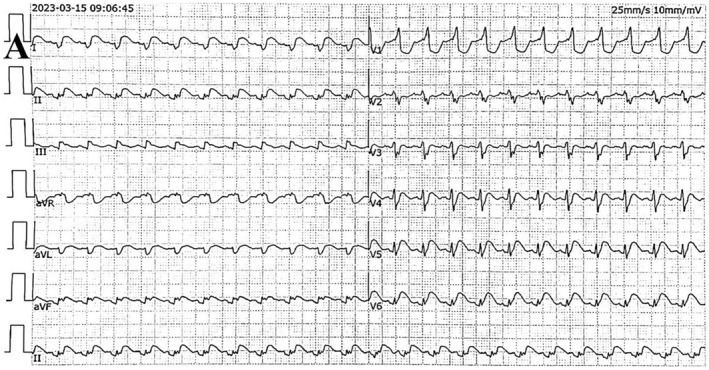
(A) Showing electrocardiogram on the first day of hospitalization.

A 28‐year‐old male (height 174 cm, weighing 55 kg) was admitted to the Emergency Intensive Care Unit (EICU) at Sichuan Province People's Hospital on March 15, 2023, due to recurrent fever over a period of 5 days. On the sixth hour of admission, ventricular fibrillation occurred abruptly in this patient. The electrocardiogram displayed ST segment elevation ranging from 0.05 to 0.2 mv (Figure 1 panel A) in leads V4 through V6 and the level of high‐sensitivity troponin T (hs‐cTnT)was measured at 5135 ng/L. Based on the examination results and clinical symptoms, the physician diagnosed fulminant myocarditis, and cardiogenic shock.

## 3 Methods

### 3.1 Application and Management of ECMO

Electric defibrillation was promptly administered with ongoing chest compression. The patient was immediately connected to VA‐ECMO with the initial speed of 9200/min, the blood flow of 3.6 L/min, the norepinephrine of 1.5 μg/(kg/min), and heparin anticoagulation of 1 μ/(kg/h). Activated coagulation time (ACT) was monitored every 4 h while activated partial thromboplastin time (APTT), prothrombin time (PT), and anti‐factor‐Xa levels (anti‐Xa) were monitored four times a day.

### 3.2 Anticoagulant Treatment

**FIGURE 2:**
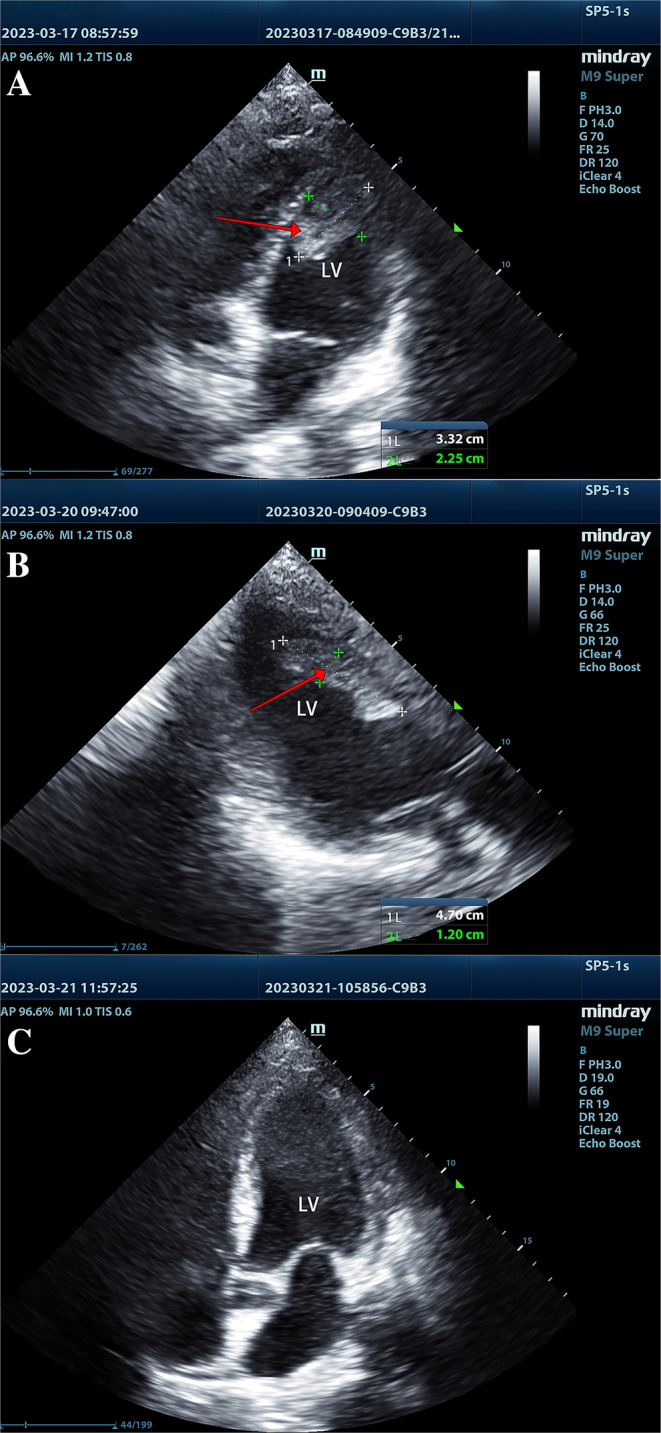
(A) Showing that on March 17, 2023, an echocardiogram revealed a left ventricular thrombus measuring 33 × 23 mm. (B) Showing that the maximum size of the thrombus (47 × 12 mm) was measured on March 20, 2023. (C) Showing that the left ventricular thrombus on March 21, 2023.

**FIGURE 3:**
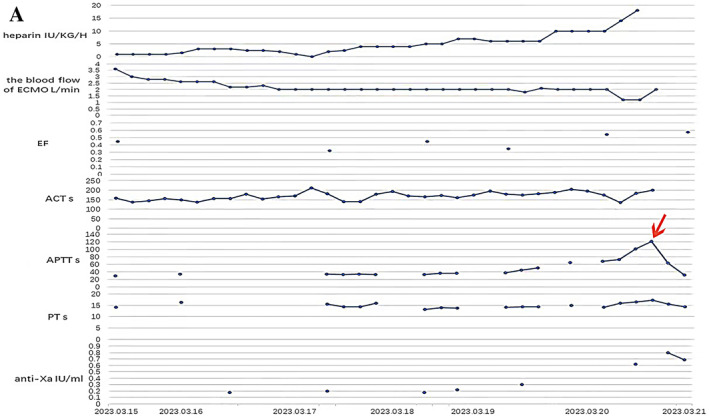
(A) Monitoring values of anticoagulant dosage, coagulation function, and partial correlation index during extracorporeal membrane oxygenation (ECMO).

On March 17, 2023, the ultrasonic cardiogram revealed a thrombus in the patient's LV, measuring 33 by 23 mm in width (Figure 2 panel A, arrow). By March 20, 2023, the thrombus had enlarged to a maximum diameter of 47 by 12 mm (Figure 2 panel B, arrow), and left ventricle ejection fraction (LVEF) was determined 54%. Due to compromised cardiac function, the surgical procedure is not an option, Therefore, we tried to apply a simple anticoagulant treatment strategy to eliminate the LV thrombus and maintaining both ACT> 180s and APTT> 60. The dosage of heparin for anticoagulation was 18 μ/(kg/h) according to the clinical examination results, and the maximum APTT was 121.5s (Figure 3 panel A, arrow).

On March 20, 2023, the patient met the criteria for weaning off ECMO. However, we encountered a challenging decision: the thrombus remains, if we removed ECMO, there was a significant risk of cardiac arrest; but if we did not, it was difficult to maintain a balance between thrombosis and bleeding while APTT is significantly prolonged, and may lead to fatal hemorrhage. After a thorough evaluation of the risks confronting the patient, we decided that the likelihood of fatal hemorrhage was significantly greater than cardiac arrest. Consequently, we opted to wean the patient from ECMO. Subsequently, the anticoagulant regimen was adjusted to enoxaparin at a dosage of 6000 IU per 12 h by subcutaneous injection.

After 5 days of treatment, there was no obvious abnormal echo in the left ventricle revealing by color Doppler echocardiography (Figure 2 panel C). On the 13th day, patient was successfully weaned off mechanical ventilation and the patient continued to receive subcutaneous injections of enoxaparin at a dosage of 3000iu per 12 h.

## 4 Conclusions and Results (Outcome and Follow‐Up)

Finally, the patient's condition improved progressively, and he was discharged on the 29th day of hospitalization without any complications. During the 3‐ and 9‐month follow‐up appointments, echocardiography revealed no significant presence of a thrombus within the left ventricle. Additionally, the patient reported no significant discomfort during the subsequent 9 months.

LV thrombosis is one of the most serious complications during ECMO. At present, the treatment of LV thrombosis during ECMO mainly includes drug therapy, surgical thrombectomy, and LV load unloading with no consensus. According to our case, we demonstrated that a feasible and practical simple anticoagulant treatment strategy can eliminate significant LV thrombosis which occurred during ECMO therapy for patient with fulminant myocarditis.

## 5 Discussion

LV thrombosis is a rare but serious complication which has a high risk of death for patients. Inadequate anticoagulation is one of the primary causes. Di Odoardo LAF et al. [10] proposed that, in the presence of LV thrombosis, low‐molecular‐weight heparin is the most used in‐hospital therapy. Furthermore, researchers have proposed employing ACT as a monitoring marker to direct anticoagulant therapy [11, 12]. However, there is no consensus on the optimal utilization of heparin, as relevant studies are insufficient to provide conclusive evidence.

We reviewed previous research of LV thrombosis during ECMO treatment. We used keywords such as “LV thrombosis” and “ECMO” to retrieve relevant literatures through PubMed (from 1993 to December 2023). Upon investigation, we found only eight case studies and one literature review [13, 14, 15, 16, 17, 18, 19, 20, 21]. When reading the case studies (Table 1), we found five patients were survived, and three deceased. As for the literature review, all patients deceased. Among the five patients that survived, the treatments vary. One of them [21] applied the Tenecteplase 30 mg by atomization. Teneplase (TNK) is a genetically engineered rt‐PA with pharmacological characteristics of single intravenous infusion, high fibrin specificity, and strong plasminogen activator inhibitor (PAR‐I) resistance. This regimen ended the 20 years dilemma where the third‐generation thrombolytic drugs could not be used on Chinese patients [22]. Three [13, 17, 18] received surgical thrombectomy, and another one [14] was treated with a new technology‐placed retrograde aortic root catheter for heparin infusion.

**TABLE 1:** Case reports of LV thrombosis during ECMO.

Year	Author	Sex	Age (years)	Cause	Thrombosis volume	Thrombosis location	Outcome
2020	Aljohani et al. [14]	M	15	Cardiogenic shock	About 1 × 0.75 cm	Ending from the LV cavity the aortic root	Survival
2018	Huerter et al. [13]	F	33	Cardiac arrest	Not described	LV, the aorta, and the superior mesenteric artery	Survival
2017	Pořízka et al. [15]	M	73	Respiratory failure	2.6 × 7.5 cm	LV	Death
2017	Ogawa et al. [17]	F	57	Cardiogenic shock	500 g	LA and LV	Survival
2015	Sangalli et al. [21]	F	54	Myocardial infarction	About 3 × 1 cm	LV	Survival
2014	Freud et al. [18]	F	5	Cardiogenic shock	About 2 × 0.5 cm	LV and the aortic valve	Survival
2013	Kuhl et al. [20]	M	49	Cardiogenic shock	4 × 4 × 2.5 cm	LV	Death
2013	Ramjee et al. [16]	M	57	Cardiogenic shock	About 3 × 3 cm	LA, LV, and proximal ascending aorta	Death

Abbreviations: F, female; LA, left atrium; LV, left ventricle; M, male.

In our study, we reported a case of the largest LV thrombus that occurred during ECMO treatment. According to existing literature, our patient is the only reported case of successful treatment for LV thrombus sequentially using heparin and low‐molecular‐weight heparin, particularly in the presence of such a large thrombus (Table 1). Compared with other studies, our antithrombotic treatment strategy is more practical, safer, and helps reduce the economic burden of patients. In addition, the patient tested positive for anti‐RO‐52 antibody and anti‐mitochondrial antibody M2 subtype upon admission. However, there is no evidence in the literature that can confidently establish a causal link between these antibodies and thrombus.

## Author Contributions

**Xue‐ting Yang:** conceptualization, resources, writing – original draft. **Yi‐jin Chen:** conceptualization, data curation, formal analysis, writing – original draft. **Han Zeng:** writing – original draft. **Lei Deng:** data curation. **Li Chang:** data curation. **Yi Li:** data curation. **Meng‐long Song:** data curation. **Yang Zhang:** data curation. **Wei Peng:** data curation. **Dong Wang:** writing – review and editing. **Hua Jiang:** conceptualization, funding acquisition, supervision, writing – review and editing. **Hong‐qiong Peng:** supervision.

## Consent

Written informed consent was obtained from the patient to publish this report in accordance with the journal's patient consent policy.

## Conflicts of Interest

The authors declare no conflicts of interest.

## Data Availability

All data and materials related to this work are available from the corresponding authors upon reasonable request.

## References

[1] E. Ammirati , G. Veronese , M. Cipriani , et al., “Acute and Fulminant Myocarditis: A Pragmatic Clinicalapproach to Diagnosis and Treatment,” Current Cardiology Reports 20, no. 11 (2018): 114–126, 10.1007/s11886-018-1054-z. 30259175

[2] B. Maisch , V. Ruppert , and S. Pankuweit , “Management of Fulminant Myocarditis: Adiagnosis in Search of Its Etiology but With Therapeutic Options,” Current Heart Failure Reports 11, no. 2 (2014): 166–177, 10.1007/s11897-014-0196-6. 24723087

[3] N. Saraiya , S. Singh , and M. Corpuz , “Fatal Influenza Myocarditis With Incessant Ventricular Tachycardia,” BML Case Reports 12, no. 7 (2019): e228201–e228203, 10.1136/bcr-2018-228201. PMC6605955 31266755

[4] P. Ponikowski , A. VoorsA , S. D. Anker , et al., “ESC Guidelines for the Diagnosis and Treatment of Acute and Chronic Heart Failure: The Task Force for the Diagnosis and Treatment of Acute and Chronic Heart Failure of the European Society of Cardiology (ESC). Developed With the Special Contribution of the Heart Failure Association (HFA) of the ESC,” European Heart Journal 18, no. 8 (2016): 891–975, 10.1002/ejhf.592. 27207191

[5] J. D. Hill , T. G. O'Brien , J. J. Murray , et al., “Prolonged Extracorporeal Oxygenation for Acute Post‐Traumatic Respiratory Failure (Shock‐Lung Syndrome). Use of the Bramson Membrane Lung,” New England Journal of Medicine 286, no. 12 (1972): 629–634, 10.1056/NEJM197203232861204. 5060491

[6] J. Ali and A. Vuylsteke , “Extracorporeal Membrane Oxygenation: Indications, Technique and Contemporary Outcomes,” Heart 105, no. 18 (2019): 1437–1443, 10.1136/heartjnl-2017-311928. 31040171

[7] Y. Peng , Y. Yao , S. Li , et al., “Meta‐Analysis of the Incidence of Extracorporeal Membrane Oxygenation Combined With Incident Thrombosis,” ClinicalFocus 39, no. 1 (2024): 5–11.

[8] A. Zangrillo , G. Landoni , G. Biondi‐Zoccai , et al., “A Meta‐Analysis of Complications and Mortality of Extracorporeal Membrane Oxygenation,” Critical Care and Resuscitation 15, no. 3 (2013): 172–178. 23944202

[9] J. H. Kim , M. Pieri , G. Landoni , et al., “Venovenous ECMO Treatment, Outcomes, and Complications in Adults According to Large Case Series: A Systematic Review,” International Journal of Artificial Organs 44, no. 7 (2021): 481–488, 10.1177/0391398820975408. 33259258

[10] L. A. F. Di Odoardo , M. Bianco , I. J. N. Gil , et al., “Left Ventricular Thrombus Management After Acute Myocardial Infarction in Clinical Practice: Results From LEVITATION Survey and Narrative Review,” Cardiovascular Drugs and Therapy 38, no. 3 (2024): 483–492, 10.1007/s10557-022-07417-w. 36538031

[11] C. W. Baird , D. Zurakowski , B. Robinson , et al., “Anticoagulation and Pediatric Extracorporeal Membrane Oxygenation: Impact of Activated Clotting Time and Heparin Dose on Survival,” Annals of Thoracic Surgery 83, no. 3 (2007): 912–920, 10.1016/j.athoracsur.2006.09.054. 17307433

[12] L. M. Ryerson , A. K. Bruce , L. Lequier , S. Kuhle , M. P. Massicotte , and M. E. Bauman , “Administration of Antithrombin Concentrate in Infants and Children on Extracorporeal Life Support Improves Anticoagulation Efficacy,” ASAIO Journal 60, no. 5 (2014): 559–563, 10.1097/MAT.0000000000000099. 24814836

[13] M. Huerter , D. Govostis , M. Ellenby , and E. Smith‐Singares , “Acute Bowel Ischemia Associated With Left Ventricular Thrombus and Arteriovenous Extracorporeal Membrane Oxygenation,” Journal of Extra‐Corporeal Technology 50, no. 1 (2018): 58–60. 29559756 PMC5848086

[14] O. A. Aljohani , R. K. Singh , S. J. Nageotte , et al., “Aortic Root Thrombosis on ECMO‐A Novel Management Strategy,” World Journal for Pediatric & Congenital Heart Surgery 11, no. 5 (2020): 643–645, 10.1177/2150135120924416. 32853069

[15] M. Pořízka , P. Kopecký , V. Mikulenka , J. Kunstýř , M. Lipš , and M. Balík , “Catastrophic Left Ventricular Thrombosis Complicating Extra‐Corporeal Membrane Oxygenation: A Case Report,” Prague Medical Report 118, no. 4 (2017): 139–146, 10.14712/23362936.2017.15. 29324221

[16] V. Ramjee , S. Shreenivas , J. E. Rame , J. N. Kirkpatrick , and D. Jagasia , “Complete Spontaneous Left Heart and Aortic Thromboses on Extracorporeal Membrane Oxygenation Support,” Echocardiography 30, no. 10 (2013): E342–E343, 10.1111/echo.12323. 23889597

[17] M. Ogawa , K. R. Balsara , M. F. Masood , and A. Itoh , “Extracorporeal Left Ventricular Circulatory Support as a Bridge to Implantable LVAD for a Patient With Pan‐Left Ventricular Thrombosis,” ASAIO Journal 63, no. 4 (2017): e45–e46, 10.1097/MAT.0000000000000432. 27556143

[18] L. R. Freud , P. R. Koenig , H. M. Russell , and A. Patel , “Left Ventricular Thrombus Formation After Repair of Anomalous Left Coronary Artery From the Pulmonary Artery,” World Journal for Pediatric & Congenital Heart Surgery 5, no. 2 (2014): 342–344, 10.1177/2150135113510185. 24668990 PMC4275842

[19] C. Weber , A. C. Deppe , A. Sabashnikov , et al., “Left Ventricular Thrombus Formation in Patients Undergoing Femoral Veno‐Arterial Extracorporeal Membrane Oxygenation,” Perfusion 33, no. 4 (2018): 283–288, 10.1177/0267659117745369. 29172999

[20] T. Kuhl , S. Wendt , G. Langebartels , A. Kröner , and T. Wahlers , “Recurrent Left Atrial and Left Ventricular Thrombosis due to Heparin‐Induced Thrombocytopenia: Case Report and Short Review,” Thoracic and Cardiovascular Surgeon 61, no. 6 (2013): 537–540, 10.1055/s-0032-1328930. 23424064

[21] F. Sangalli , G. Greco , L. Galbiati , F. Formica , S. Calcinati , and L. Avalli , “Regional Thrombolysis With Tenecteplase During Extracorporeal Membrane Oxygenation: A New Approach for Left Ventricular Thrombosis,” Journal of Cardiac Surgery 30, no. 6 (2015): 541–543, 10.1111/jocs.12556. 25940057

[22] Y. Wang , S. Li , Y. Pan , et al., “Tenecteplase Versus Alteplase in Acute Ischaemic Cerebrovascular Events (TRACE‐2): A Phase 3, Multicentre, Open‐Label, Randomised Controlled, Non‐Inferiority Trial,” Lancet 401, no. 10377 (2023): 645–654, 10.1016/S0140-6736(22)02600-9. 36774935

